# A Bottom-Up Validation of the IAPS, GAPED, and NAPS Affective Picture Databases: Differential Effects on Behavioral Performance

**DOI:** 10.3389/fpsyg.2020.02187

**Published:** 2020-09-04

**Authors:** Michela Balsamo, Leonardo Carlucci, Caterina Padulo, Bernardo Perfetti, Beth Fairfield

**Affiliations:** Department of Psychological, Health and Territorial Sciences, University of Chieti, Chieti, Italy

**Keywords:** emotion, attention, International affective picture system, Geneva affective picture database, Nencki affective picture system

## Abstract

The concept of emotion is a complex neural and psychological phenomenon, central to the organization of human social behavior. As the result of subjective experience, emotions involve bottom-up cognitive styles responsible for efficient adaptation of human behavior to the environment based on salient goals. Indeed, bottom-up cognitive processes are mandatory for clarifying emotion-cognition interactions. Accordingly, a huge number of studies and standardized affective stimuli databases have been developed (i.e., International Affective Picture System (IAPS), Geneva Affective Picture Database (GAPED), and Nencki Affective Picture System (NAPS)). However, these neither accurately reflect the complex neural system underlying emotional responses nor do they offer a comprehensive framework for researchers. The present article aims to provide an additional bottom-up validation of affective stimuli that are independent from cognitive processing and control mechanisms, related to the implicit relevance and evolutionistic significance of stimuli. A subset of 360 images from the original NAPS, GAPED, and IAPS datasets was selected in order to proportionally cover the whole dimensional affective space. Among these, using a two-step analysis strategy, we identified three clusters (“good performance”, “poor performance”, and “false alarm”) of stimuli with similar cognitive response profiles. Results showed that the three clusters differed in terms of arousal and database membership, but not in terms of valence. The new database, with accompanying ratings and image parameters, allows researchers to select visual stimuli independent from dimensional/discrete-categories, and provides information on the implicit effects triggered by such stimuli.

## Introduction

There is unanimous agreement that the complexity of human feelings and the concept of emotion are complex neural and psychological phenomena, central to the organization of human behavior. Accordingly, emotion has been widely investigated and psychologists have advanced more than a single definition aimed to highlight specific aspects. A recent review (Izard, 2010) focusing on the commonalities among the diverse definitions, defined emotion as the result of subjective experience, variations in physiological state and behavioral outcomes, and strengthening the idea that emotion prompts an organism to act in response to and consistent with environmental demands (Inzlicht et al., 2015). This definition clearly links emotion to cognition and cognitive control (i.e., the mental processes responsible for efficient adaptation of human behavior to the environment based on salient goals), suggesting that emotion and cognition are integrated and consequently can have reciprocal selective effects (Gray, 2004). Indeed, this strong connection has become the focus of a huge number of studies evaluating the reciprocal effects on different facets of emotion and cognition including memory, attention, and executive control, across a variety of tasks and different stimuli. In addition, the emotion cognitive interaction involves both bottom-up and top-down pathways of human information processing. For example, on the one hand, orienting spatial attention relies on top-down mechanisms initiated to select information for further processing according to individual goals and the task at hand. On the other, spatial attention can also be attracted by salient and/or potentially dangerous events *via* bottom-up mechanisms in response to unexpected but important events.

As a result, careful selection of controlled emotion stimuli is crucial for inducing and/or investigating the constructs under investigation. This study aims to classify emotion stimuli according to their effects in terms of hits, false alarms, and reaction times (RTs) on attention performance. This may allow researchers to select the best exemplars and to discriminate bottom-up mechanisms and clarify emotion-cognition interactions. In particular, although emotionally charged stimuli in different modalities (e.g., auditory, lexical, and visual) have been adopted in both behavioral and neuroimaging research (Zeelenberg and Bocanegra, 2010; Brooks et al., 2012; Sylvester et al., 2016), the visual channel is probably the most common. In the visual channel, emotional stimulation has been achieved through movie presentation (Gross and Levenson, 1993), complex images, or meaningful faces (Lang et al., 1993; Codispoti et al., 2001; Stark et al., 2004) and researchers can rely upon many sets of standardized items together with measures along various axes fundamental to emotion. Among these sets, the gold-standard of emotionally charged visual complex pictures is probably the International Affective Picture System (IAPS), developed by Lang and colleagues (Center for the Study of Emotion and Attention; Lang et al., 1988, 1997, 1999, 2001). In this database, each item is accompanied by a series of norms (means and standard deviation) along three dimensions: arousal (physiological activation evoked by the image), valence (pleasantness and pleasure), and dominance (the degree to which the emotional state is under subject’s control). This set has also been standardized according to the dimensional-category theory of emotion which holds that affective experiences can be characterized by the above-mentioned dimensions as well as the approach-avoidance dimension (Mauss and Robinson, 2009). More recently, the IAPS has been standardized according to a discrete-category theory of emotion that proposes at least five basic universal emotions. Indeed, discrete-category theories of emotion assume that traditional dimensions are too simple and therefore, do not accurately reflect the complex neural system underlying emotional responses (Darwin and Prodger, 1998; Mauss and Robinson, 2009).

In line with discrete-category theories of emotion, other affective static image databases with various content and validated normative ratings have been developed. To date, the most internationally recognized databases are the Geneva Affective Picture Database (GAPED; Dan-Glauser and Scherer, 2011) and the Nencki Affective Picture System (NAPS; Marchewka et al., 2014). The former includes negative pictures depicting four specific categories (i.e., spiders, snakes, and scenes that induce emotions related to human rights violations or animal mistreatment), neutral pictures that mainly represent objects, and positive pictures that mainly depict human and animal babies and natural scenery. Valence, arousal, internal (moral), and external (legal) norms have been collected for the images in this discrete-category organization. The latter, instead, provides high-quality images organized in five discrete categories (i.e., people, faces, animals, objects, and landscapes) that have been evaluated, using semantic bipolar scales, for arousal, valence, and motivational direction (i.e., approach-avoidance dimension).

However, when individuals are asked to judge stimuli from a database, they must deeply elaborate each stimulus in order to formulate an appropriate affective judgment. In this manner, they rely on top-down controlled cognitive resources that are crucial for making affective judgments. Yet, it is widely recognized that brain structures linked to the processing of affective information are often subcortical (e.g., amygdala, ventral striatum, and hypothalamus). Moreover, these structures are considered primitive and operate in a fast and automatic fashion. It follows that certain “trigger” features are relatively unfiltered and evoke responses that might be important for survival. Indeed, an individual need not be conscious of the trigger stimulus (e.g., the white of eyes in a fearful expression) that elicited activation in an affective brain region.

Extensive literature has examined the link between affective information and cognition and has shown how affective stimuli can directly modulate cognitive performance through bottom-up processes such as attention orientating, and consequently memory (Murphy and Isaacowitz, 2008; Talmi et al., 2008; Brenner et al., 2014; Padulo et al., 2020). More specifically, the boost in sensory processing for emotionally salient events (Vuilleumier, 2002, 2005), enhances attention toward them and/or alters attention toward other concomitant stimuli (Dennis and Chen, 2007; Bocanegra and Zeelenberg, 2009; Vuilleumier and Huang, 2009) and ultimately leads to more efficient encoding and consolidation in memory. In this manner, bottom-up processing of affective stimuli orients attention and subsequently engages emotional processing mechanisms that rely on more top-down cognitive processes.

Here, we aimed to provide an additional bottom-up validation of the above-mentioned picture databases that can be consulted when choosing affective stimuli for an experimental paradigm. We used existing norms to select the best exemplars from each database with the intent to cover all the emotional dimensions. To elicit implicit effects linked to the interaction between emotion and attention orienting, we used the dot-probe task, in which attention is modulated by the presentation of two task-irrelevant cues before probe presentation. In this task, attention is automatically captured by one of the formers based on both the relative salience and the congruency between attended cue and probe locations, leading to differential behavioral responses (Bradley and Lang, 1999, 2000; Carrasco et al., 2004; Bradley, 2009). We postulated at least three main advantages of our bottom-up validation: (1) it should be independent from cognitive processing and control mechanisms necessary when formulating appropriate affective judgments, (2) it should be closely related to the implicit relevance and evolutionistic significance of stimuli, and (3) it should be independent from dimensional-category and discrete-category theoretical background, and provide information on the implicit effects triggered by such stimuli.

## Materials and Methods

### Participants

Participants included 199 young adults (33 males), with a mean age of 21.28 (*SD* = 4.47; range: 19–27) years and with a mean education of 14.89 (*SD* = 1.35) years. All volunteers were recruited from the University of Chieti-Pescara and compensated with class credit. All participants were right-handed, native Italian speakers, had normal or corrected normal vision, with no reports of psychiatric or neurological disorders, use of psychiatric drugs, or any medication that could potentially interfere with their mental processing. Participants signed informed consent forms approved by the Department of Psychological Sciences, Health and Territory, University of Chieti, Italy, Review Board before taking part in the experimental session. Participants were randomly sorted into three different groups. Each group executed the same attentional task but with a different set of affective images drawn from one of three databases described above (IAPS, NAPS, and GAPED). Before beginning the experimental task, all participants underwent cognitive, mood, and personality evaluations. General cognition was assessed with the Culture Fair Intelligence Test (CFIT) Scale 3 (Cattell and Cattell, 1959). We administered the “State Trait Inventory of Cognitive and Somatic Anxiety” (STICSA) for mood (Balsamo et al., 2015; Carlucci et al., 2018) to assess cognitive and somatic anxiety symptoms and consisting of both trait and state versions; and the “Teate Depression Inventory” (TDI; Balsamo and Saggino, 2013) to detect major depressive disorder as specified by the latest edition of the Diagnostic and Statistical Manual of Mental Disorders (DSM-V). We evaluated personality traits with the short form of the “Big Five Questionnaire” (BFQ; Caprara et al., 1993) with five general domain scales (energy, agreeableness, conscientiousness, emotional stability, and openness).

All questionnaires were given *via* web (Qualtrics software; Qualtrics, Provo, UT). The cognitive, mood, personality and demographic variables are reported in Table 1 for the three groups separately along with the statistics testing possible differences among samples.

**Table 1:** Descriptive statistics of cognitive, mood, personality, and demographic variables.

	Group			ANOVA	
	IAPS (*N* = 67) mean (SD)	GAPED (*N* = 66) mean (SD)	NAPS (*N* = 66) mean (SD)	*F*	Sig.
Age	21.25 (1.90)	20.74 (1.88)	20.64 (1.63)	2.22	0.11
School	15.16 (1.24)	14.86 (1.47)	14.64 (1.31)	2.60	0.08
Culture fair	24.66 (4.21)	24.72 (4.19)	24.65 (4.11)	0.01	0.99
TDI	2.43 (0.52)	2.52 (0.46)	2.46 (0.53)	0.52	0.60
STICSA-trait	35.81 (8.13)	35.70 (7.35)	35.82 (7.03)	0.01	0.99
STICSA-state	31.45 (6.92)	32.68 (6.91)	32.45 (7.18)	0.58	0.56
BFQ-extraversion	78.18 (12.62)	75.64 (12.89)	75.42 (12.41)	0.98	0.38
BFQ-consciousness	90.84 (14.28)	91.79 (13.63)	89.79 (14.62)	0.33	0.72
BFQ-neuroticism	64.78 (16.91)	62.85 (17.49)	64.06 (16.75)	0.22	0.81
BFQ-agreeableness	85.70 (13.41)	83.52 (10.74)	83.06 (9.59)	1.03	0.36
BFQ-openness	90.96 (11.24)	86.88 (12.8)	87.82 (13.64)	1.91	0.15
		**Group**			**Total**
		**IAPS**	**NAPS**	**GAPED**	
Gender	F	57	55	54	166
	M	10	11	12	33
	Total	67	66	66	199

STICSA, State Trait Inventory for Cognitive and Somatic Anxiety; BFQ, Big Five Questionnaire.

### Images Selection

We selected a total of 360 images from the IAPS and NAPS datasets (180 stimuli each; 60 negative, 60 neutral, and 60 positive) and 168 items from the GAPED (56 negative, 56 neutral, and 56 positive). Image selection was based on specific criteria taking into consideration both the dimensional (valence and arousal) and discrete (i.e., happiness, anger, fear, etc.) ratings available for the sets intended to maximize the differences among the three affective categories (negative, neutral, and positive). Specific details for each database are as follows.

Images for the IAPS were selected on the original norms and their updates (Lang et al., 1997, 2008; Libkuman et al., 2007). Briefly, the criteria for including pictures in the current study were: (1) mean valence ratings for unpleasant stimuli <25th percentile on both datasets and >75th percentile on the “anger and fear” dimension; mean standard deviation scores for negative between 25 and 75th percentile on both norms; (2) valence ratings for the pleasant images >75th percentile on both datasets and scores on the “happiness” dimension >75th percentile; mean standard deviation scores for positive stimuli between 25 and 75th percentile on both norms; and (3) neutral stimuli within the range of mean ± 1/3 SD in both datasets. In order to keep the image number within the pre-established range, further selection was based on valence: we selected the first 60 images with lowest ratings for the unpleasant category, the first 60 pictures with the highest scores for the pleasant category and the 60 images closest to the mean for the neutral category, respectively.

In addition, to maximize differentiation between stimuli, we used an important characteristic of the GAPED dataset. This dataset adopts a topic-oriented approach, with selection of the pictures mainly driven by their affective content, yielding a range of images with high biological, evolutionary, and social relevance. In this study, we selected 168 images from the original GAPED norms (Dan-Glauser and Scherer, 2011) based on the following criteria: (1) mean valence ratings for unpleasant stimuli <25th and mean standard deviation scores between 25 and 75th percentile. This yielded a set of 74 items drawn from the human rights violation and animal mistreatment categories; (2) based upon authors’ considerations (Dan-Glauser and Scherer, 2011) and on electrophysiological data showing attention-orienting responses driven by positive nurturance-relevant stimuli, we decided to select the positive pictures from the set of images representing human babies and young animals. Selection resulted in a set of 56 images. (3) Neutral stimuli within the range of mean ± 1/2 SD resulting in 63 items. In order to have consistency among the positive, negative and neutral categories in terms of set size, we limited the number of images to the minimum size obtained by our selection. Hence, 56 positive and 56 neutral pictures were randomly drawn from our selection.

Selection of the 180 images from the NAPS followed the criteria already reported for the IAPS. First, mean valence ratings under the 25th percentile and above the 75th for negative and positive items, respectively. In addition, positive and negative items were included between the 25 and 75th percentile on the distribution of valence standard deviation. Interestingly, the selection on valence ratings resulted in a pool of negative items equally distributed among fear, sadness, and disgust categories, while positive items had high score on happiness and surprise categories. Finally, neutral stimuli had mean valence scores between the mean and ±1/3 SD.

### Experimental Setting

An Intel-based computer running E-Prime 2 (Psychology Software Tools, Pittsburgh, PA) controlled stimuli presentation and data acquisition. Stimuli were presented visually on a 15.2-inch computer screen. During the entire experiment, a white fixation cross (0.5° of visual angle) and two white rectangular frames (4° × 5.17° visual angle; 7 × 10 cm) appeared on a gray background. Fixation was located at the center of the screen while the two rectangular frames were presented in the left and right upper visual fields. Inner edges of the latter were 6°‐ horizontally and 3°-visual angles vertically apart from the fixation cross. Affective colored images and target stimuli were presented inside the white frames. Target stimuli consisted of circular Gabor patterns modulated either horizontally or vertically in black and white that had a diameter of 4° visual angle and a spatial frequency of 4 cycles per degree. Responses to targets were acquired through a computer keyboard. Participants were seated approximately 100 cm from the screen.

### Experimental Task and Procedures

**Figure 1:**
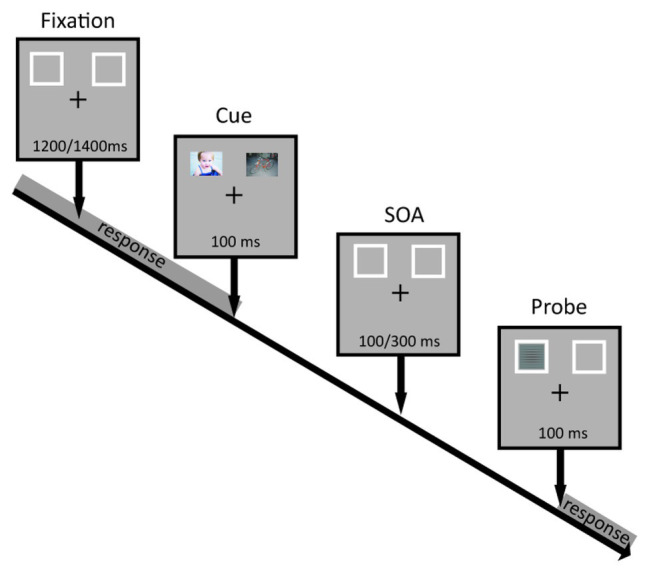
Visualization of the experimental stimuli and procedure.

Each group of participants executed the same attentional task but with a different set of affective images from one of the three databases (IAPS, NAPS, and GAPED). We adopted a modified version of the dot-probe task to evaluate the orienting of attention as a function of the emotional valence. Specifically, we presented three types of image pairs: negative-neutral, positive-neutral, and negative-positive. An example of a trial is presented in Figure 1. Each trial started with a fixation cross and the two rectangular frames, one in the left and one in the right visual field (LVF and RVF, respectively) presented randomly between 1,200 and 1,400 ms (in 50 ms steps). Immediately after, two affective images were presented simultaneously inside the two frames for 100 ms followed by a variable interval (100, 150, 200, 250, and 300 ms). After the delay, a target stimulus appeared in either the left or right rectangular frame for 100 ms. The experimental session consisted of six blocks for a total of 720 trials. Participants were instructed to respond only to targets having either horizontal or vertical orientation (depending on the initial instructions) as quickly as possible by pressing the space bar with their right index finger. Of note, we manipulated the shape of the fixation cross so that either the horizontal or vertical branch was thicker than the other. In this manner participants were reminded of which judgment to make to avoid confusion between blocks. We asked participants to fix on the central cross throughout the whole experimental session, and to covertly pay attention to the visual stimuli presented laterally. In addition, participants were explicitly told that the images preceding the target were not informative of its location.

All participants completed a practice session consisting of a total of 20 trials to familiarize with the task. Only participants who completed at least 90% of trials correctly took part in the experimental session. We manipulated emotional attention by presenting image pairs according to three different experimental conditions: negative-neutral, positive-neutral, and negative-positive. Each block consisted of 40 image pairs per condition (20 in the RVF and 20 in the LVF). Half of the targets appeared in the RVF. For these 10 items, five corresponded to the judgment to make (for example vertical; valid) while the other five did not correspond to the judgment for that block (for example horizontal; invalid). The other half of the targets appeared in the LVF. For these 10 items, five again corresponded to the judgment to make (for example vertical; valid) while the other five did not correspond to the judgment for that block (for example horizontal; invalid). Each stimulus appeared four times within the entire task.

For each image pair we collected RTs, Hits (the correct detection of a target), and False Alarms (yes-response to non-targets). These were computed by averaging the indices among subjects within each group (IAPS, GAPED, and NAPS), and separately for image pair (i.e., neutral-negative, neutral-positive, and negative-positive) and target type (valid and invalid). Given the aim of the study (characterization of a subset of images based on their capability to capture attention resources), we focused only on valid trials.

### Analysis

Data analysis was carried out in two steps. First, we explored possible differences between images from the three databases to identify potential confounding variables that might have biased performance on the attention task. Specifically, we portrayed the distribution of the items in the affective space and tested variation in item valence and arousal scores as a function of database (IAPS, GAPED, and NAPS). Moreover, we evaluated how item luminance influenced task performance and verified whether images selected from the three datasets were associated with specific performance patterns on the attentional task. Second, we classified selected items based on the ability to modulate automatic attention in line with the hypothesis that similar patterns of performance maybe associated with groups of items that share some commonalities. To this end, attention indices associated with each item were entered in multivariate classification methods to obtained groups of stimuli able to elicit comparable cognitive patterns.

## Results

### Items Description

**Figure 2:**
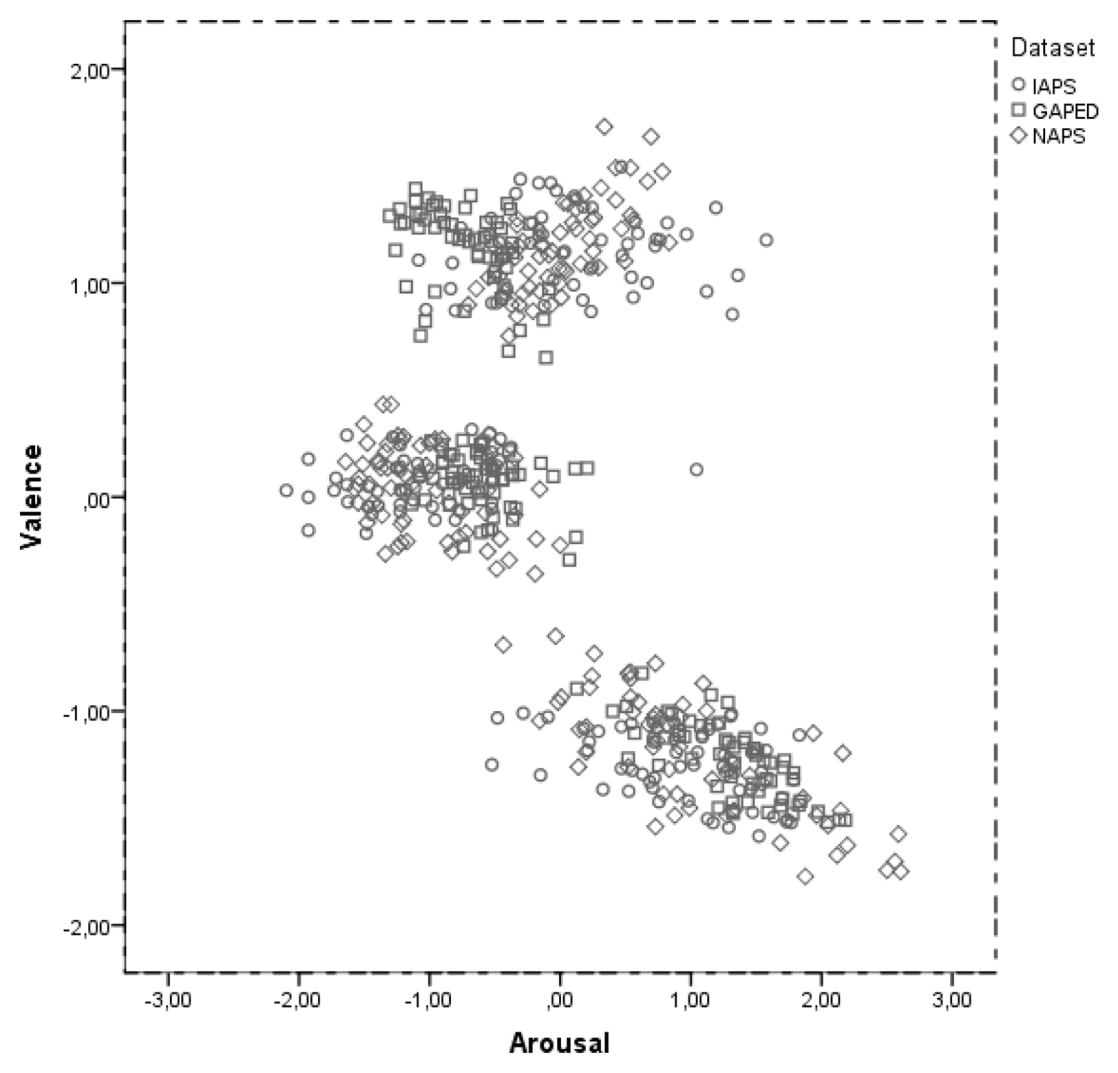
Scatterplot of the affective space for the selected items.

Figure 2 reports a scatterplot of the affective space for the selected items as a function of the original dataset. Valence and arousal scores were first standardized on their original norms. In line with previous studies (Lang et al., 2001; Libkuman et al., 2007), we observed a C shape distribution of the data. However, we obtained a more defined pooling of the items as a result of the selection procedure (maximizing the differences between items valence). At a first glance, items distribution in the affective space seems analogous among the three datasets. This was confirmed statistically by entering valence and arousal z scores into a 3 × 3 General Linear Model with Dataset (IAPS, GAPED, and NAPS) and item affective categories (neutral, negative, and positive) as factors. As expected, we found a significant effect of item affective category on valence (*F*_(2,520)_ = 6643.965; *p* < 0.001; *η_p_*^2^ = 0.963) and arousal (*F*_(2,520)_ = 673.820; *p* < 0.001; *η_p_*^2^ = 0.723). Most importantly, we did not find any reliable difference between datasets (valence, *F*_(2,520)_ = 0.011; *p* = 0.989; *η_p_*^2^ = 0.0; arousal, *F*_(2,520)_ = 0.001; *p* = 0.999 *η_p_*^2^ = 0.0).

In line with our aim to differentiate stimuli for bottom-up characteristics that can influence affective information processing, we investigated item luminance, a characteristic known to influence task performance. Indeed, visual attention is biased by visual luminance (Maunsell and McAdams, 2000; Reynolds and Desimone, 2003), and the three datasets differed along this dimension. We first computed luminance for each image by using an *ad hoc* MATLAB function (Math-Works; MA, United States), and then entered the values in a between-group ANOVA. We found that IAPS images had significant lower luminance values compared to both GAPED and NAPS (main factor effect ‐ *F*_(2,520)_ = 55.893; *p* < 0.001; *η_p_*^2^ = 0.178; *Post hoc p* < 0.001, Bonferroni corrected). However, when we evaluated the relation between luminance and the other variables of interest (item arousal and valence as well as the performance indices at the attention task), we observed small r correlations (Pearson’s) ranging from −0.114 to 0.123.

**Figure 3:**
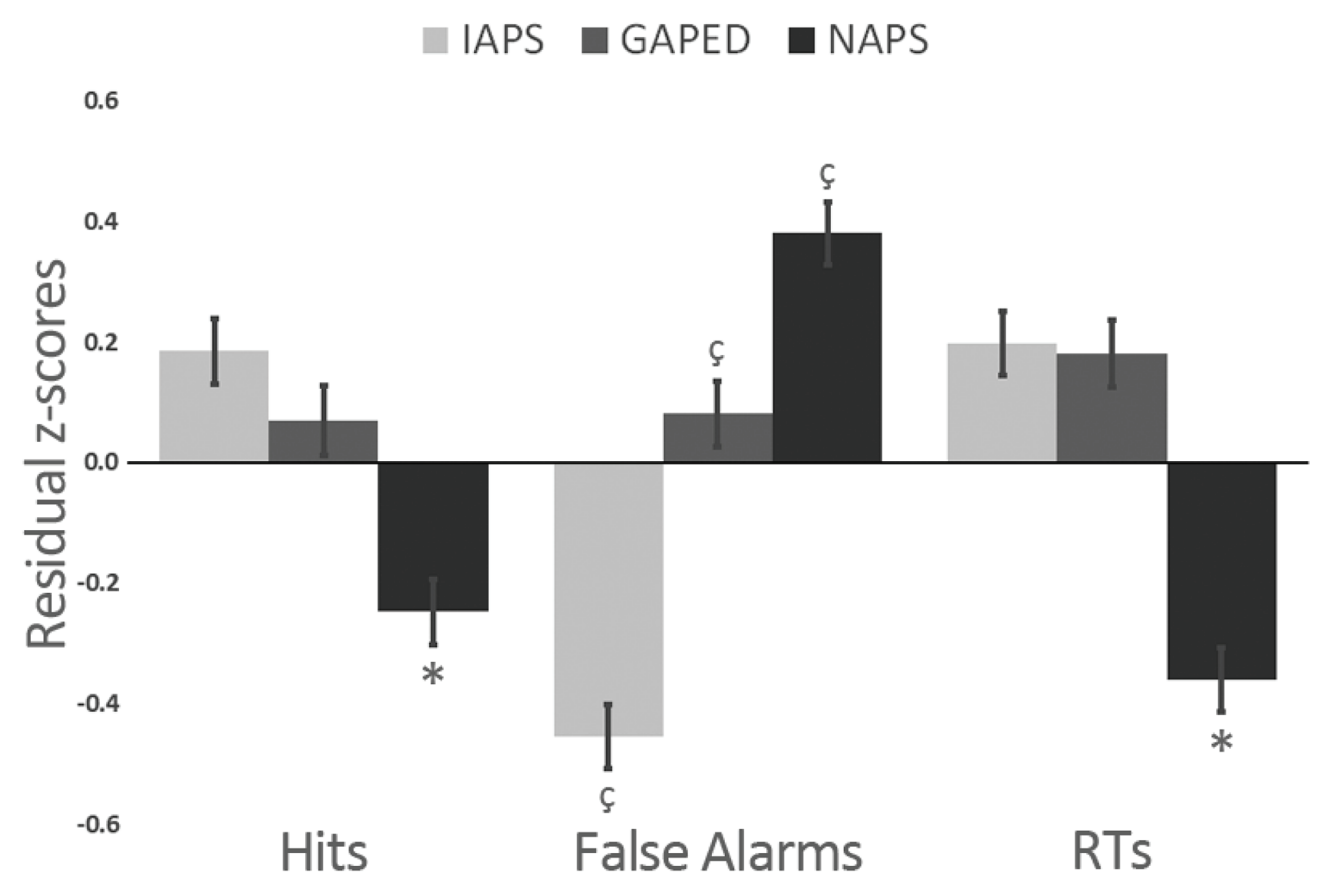
Differences between Nencki affective picture system (NAPS), Geneva affective picture database (GAPED), and International affective picture system (IAPS) dataset on Hits, False Alarms, and reaction times (RTs).

After, we proceeded by verifying whether IAPS, GAPED, and NAPS images had some intrinsic characteristics that might capture attention resources differently. To partialize out the contribution of item luminance on performance, we first ran a series of regression procedures between luminance and the behavioral task outcomes (RTs, False Alarms, and Hits) to obtain residual scores that were further entered in a 3 × 3 General Linear Model with Dataset (IAPS, GAPED, and NAPS) and Item Affective Categories as factors (neutral, negative, and positive). The only significant finding was a differences across datasets for all the three tested variables (Hits: *F*_(2,945)_ = 16.814; *p* < 0.001; *η_p_*^2^ = 0.035; False Alarm: *F*_(2,945)_ = 65.848; *p* < 0.001; *η_p_*^2^ = 0.123; RTs: *F*_(*2,945*)_ = 34.463; *p* < 0.001; *η_p_*^2^ = 0.069). Figure 3 reports findings. NAPS items were associated with less accuracy in target detection as well as faster responses compared to GAPED and IAPS. The three datasets all differed in eliciting false alarms since NAPS produced more false alarms than GAPED and IAPS, and GAPED more than IAPS.

### Cluster Analysis

We also carried out a further analysis on mean Hits, False Alarms, and RTs cognitive dimensions, a dual process clustering procedure (using hierarchical and non-hierarchical methods; see Hair et al., 1995; Bigné and Andreu, 2004), in order to identify subgroups of stimuli with similar cognitive responses profile. Thus, no a-priori number of clusters was specified. Of note, as for the previous analysis, we used z-scores (*M* = 0 and *SD* = 1) to yield equal metrics and equal weighting.

We performed the hierarchical clustering analysis in exploratory way, using the squared Euclidean distance matrix with Ward’s linkage method (Everitt et al., 1993) for forming clusters. Since there is no formal stopping rule for hierarchical cluster analysis, a cut-off point was determined according to the dendrogram to signify when the clustering process should be stopped (Bratchell, 1989). Next, in order to determine the number of clusters, information from both the agglomeration table and the dendrogram were incorporated. Results suggested a four-cluster and a three-cluster solutions, respectively. Next, the K-means cluster algorithm was applied to improve results from the hierarchical procedures and to provide more accurate cluster membership.

Using the initial seed points from the results in the hierarchical cluster, the K-means cluster defined three groups. Table 2 shows the final cluster centers. For each cluster, the mean value (centroid) is provided. In absolute terms, clusters were dissimilar, ranging from 2.44 (cluster 1 vs. 2) to 1.83 (cluster 2 vs. 3). The greater the distance between two clusters, the greater the differences in these clusters. The first cluster contained *N* = 197 images, the second *N* = 296, whereas the third *N* = 452.

**Table 2:** Composition of the final cluster centers solution.

	Cluster		
	1	2	3
Hits	−1.237	0.187	0.444
False alarm	−0.005	1.072	−0.672
RTs	1.065	−0.595	−0.093
*N images*	197	296	452

**Figure 4:**
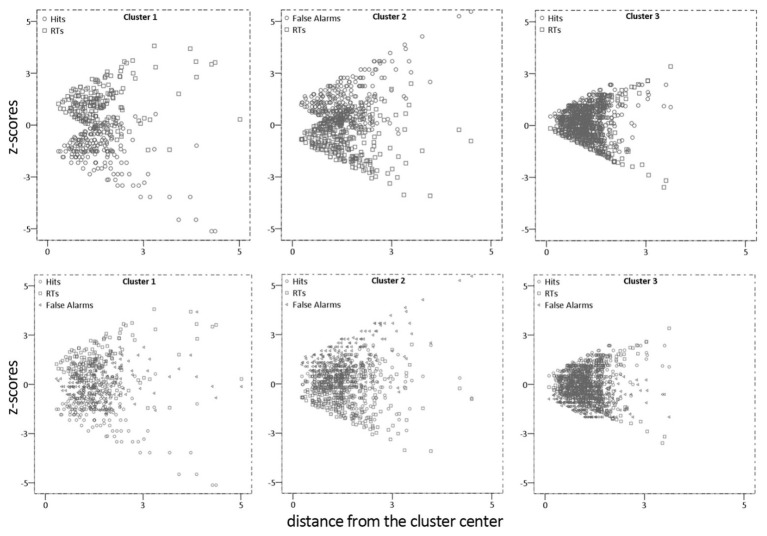
Scatterplot of distance from the center of the clusters.

In detail, the first cluster contained images with negative values of accuracy, positive values of response time as well as false alarms close to the mean. This cluster was characterized by stimuli associated with “poor performance” (i.e., low rates of accuracy and longer time to target detections). The second cluster contained images with high rate of “false alarms” and fast reaction time. Finally, the third cluster contained images that prompt “good performance,” fair accuracy, few false alarms and average response times. Results suggest that it is feasible to group affective stimuli based on patterns of cognitive performance. Figure 4 shows the distance of the items from the center of the cluster for Hits, FAs, and RTs.

### Clusters Description

After pooling items for cluster membership, we ran a set of analyses aiming at exploring the new classification. First, we investigated the affective nature of the three clusters by entering item valence and arousal scores in a Multivariate ANOVA (MANOVA) with cluster membership as factor. We found a slight effect of valence (*F*_(2,945)_ = 3.021; *p* = 0.049; *η_p_*^2^ = 0.006) that did not survive to *post hoc* procedures (all *p* > 0.05, Bonferroni corrected). The non-parametric procedure testing differences in the distribution of neutral, negative, and positive items among the three clusters found revealed no significant effect (Pearson *χ*^2^ = 6.081, *p* = 0.193). On the contrary, the three clusters differed in terms of arousal (*F*_(2,945)_ = 3.634; *p* = 0.027; *η_p_*^2^ = 0.008). Images belonging to the “poor performance” cluster were more arousing than the images in the “false alarm” cluster (*p* = 0.023, Bonferroni corrected). No statistical differences were found between images belonging to the “good performance” cluster compared to the remaining clusters (“poor performance,” *p* = 0.433; “false alarm”, *p* = 0.318). The average value close to zero displayed by the “good performance” cluster images (mean = 0.009) suggested the non-arousing effect of these images.

Second, we verified a possible association among clusters and databases. We found an unequal item distribution (Pearson *χ*^2^ = 6118.48, *p* < 0.001). Specifically, the first cluster contained images drawn equally from the IAPS, GAPED, and NAPS databases. The second cluster consisted mainly of NAPS images (54.4%): The third cluster pooled a great number of IAPS images (67.1%).

Third, we verified whether clusters could be explained by the experimental condition in which the image was presented (neutral-negative, neutral-positive, and negative-positive). As expected, we did not find any significant result (Pearson *χ*^2^ = 3.637, *p* = 0.457).

Finally, for each cluster we identified the best images based on the distance from the centroids. Quartiles were identified for the three clusters and items labeled accordingly.

Appendix 1 provides cluster membership along with its corresponding quartile, all the indices extrapolated from the attention task, the standardized valence and arousal ratings (mean and SD) obtained from the original norms, and the task condition in which it was presented for each image.

Of note, we ran the very similar cluster analysis on the same behavioral variables after removing the contribution of item luminance. The results were comparable with those reported above as for cluster membership and composition. However, we found that the small differences reported in arousal and valence across clusters disappeared, suggesting that luminance might have contributed to the findings.

## Discussion

Emotion cognition interactions are crucial for the organization of human behavior and have reciprocal effects on memory, attention, and executive control. Moreover, these interactions vary across a variety of tasks and stimuli and can be linked to both bottom-up and top-down pathways of information processing. In addition, these strategies could be biased by the individual’s affective state. For example, depressed subjects exhibited a bottom-up impairment in emotional processing (Fales et al., 2008).

Accordingly, careful stimuli selection is crucial when selecting stimuli to be used in an experimental situation or training sessions of cognitive rehabilitation with special populations. The principal aim of our study was to provide new image indexes that rely upon emotional relevance and saliency. As postulated, our analyses demonstrated that IAPS, GAPED, and NAPS images can be categorized and selected based on three main categories: (1) a cluster of “poor performance,” constituted by pictures associated mainly with low rates of accuracy and longer time to target detections, (2) a cluster of “false alarm,” composed of pictures associated mainly with high rate of false alarms and fast RTs, and (3) a cluster of “good performance” constituted by pictures associated mainly with fair accuracy rates, low false alarms and an average response time. In this way, we produced an empirical bottom-up validation of the three picture databases, already validated for valence and arousal that can be consulted along with valence and arousal ratings when choosing affective stimuli for an experimental paradigm.

Although our image section considered both dimensional (valence and arousal) and discrete (i.e., happiness, anger, fear, etc.) ratings available for the sets, so as to maximize the differences among the three affective categories (negative, neutral, and positive), we found that cluster nature is independent from valence and experimental conditions. Moreover, we found no differences in the distribution of neutral, negative, and positive items among the three clusters, so that the three picture types are well distributed among all clusters. Thus, we could argue that this new validation approach is effective in adding new and useful information for the selection of the best exemplars, considering their capability to capture and direct attention.

Regarding arousal, we found arousal was higher in the “poor performance” cluster. According to the arousal-biased competition (ABC) model (Mather and Sutherland, 2011), emotional arousal of both positive and negative stimuli amplifies the effects of bottom-up salience during visual encoding, increasing selective attention to salient stimuli. This interaction of arousal and salience may underlie the poorer performance of our sample in the dot-probe task. This may seem to be in contradiction to the ABC model, but relation between arousal and saliency and their effects on cognitive tasks are more complex. In fact, Lee et al. (2015) indicated that the arousal enhancement of cognitive functions, such as memory for previously encoded items, depends on the goal relevance initially assigned to those items. In our study, the emotional pictures were task-irrelevant cues (i.e., they were not predictors of target positions); indeed, as the level of arousal increased, and consequently salience, attention was automatically captured by pictures, leading to poorer performance in the dot-probe task. This outcome suggests that the effects that these emotional pictures can exert could be related to their implicit relevance and significance in influencing allocation of attention (Gable and Harmon-Jones, 2008, 2010; Becker, 2009; Padulo et al., 2020). Such findings corroborated the usefulness of our pictures categorization as an additional guide allows researchers to select affective images not only on affective dimensions, but also considering the effects arising from their bottom-up implicit features.

Surprisingly, although we did not find differences in the distribution of neutral, negative, and positive items among the three clusters, we found disparities in the distribution of pictures of each database among clusters. In fact, results evidenced that the “good performance” cluster consisted mainly of IAPS images whereas the “false alarm” cluster is composed mainly of NAPS images.

Despite the drawbacks, evidence in the neuroscience literature supported the high impact of IAPS images into manipulated emotional states (Liberzon et al., 2003; Hajcak et al., 2010; Beatty et al., 2014), as well as an high rate of accuracy (Britton et al., 2006). In addition, the IAPS database included a wide range of scene categories (landscapes, sexual interactions, peoples, etc.) that statistically increase the chance of detecting stimuli with good performance characteristics.

Next, a high number of the negative NAPS (*N* = 51) images were located in the “false alarm” cluster, compared to GAPED (*N* = 28), and IAPS database (*N* = 10). This datum also replicated the findings according to which negative stimuli results more frequently induced false memories than neutral and positive stimuli (Brainerd et al., 2008; Norris et al., 2019), and consequently resulted in higher rates of false alarms (Bisby and Burgess, 2014). Hypothetically, negative stimuli were encoded with gist compared to verbatim representations. This could increase the false alarm rates (Matsumoto and Kawaguchi, 2020).

It is widely known how visual attention can also be biased by visual luminance, as well as luminosity may influence image processing and subsequent memory performance (Einhäuser and König, 2003; Proulx and Egeth, 2008; Keil et al., 2013). The presence of a luminance effect on IAPS images (low luminance values) is in line with the current literature (Meiselman, 2016). IAPS images has been labeled as outdated compared the modern standards of pictures quality (i.e., brightness, contrast, and color composition; Lakens et al., 2013; Meiselman, 2016).

Unexpectedly, the luminance effect was found not to directly affect performance indices as rated by subjects in the present attention task. To date, based on the previous similar studies (Sterzer et al., 2005; Attar et al., 2010), and in order to provide a reliable and valid database norm, the contribution of item luminance on performance has been partialized out through a series of regression procedures between luminance and the behavioral task outcomes.

Limitations of this study concern statistical approach applied to select affective stimuli, sample characteristics, and stimuli characteristics considered. Despite clusters models approach represent a consolidated approach to detect similarity and dissimilarity among latent constructs in psychological literature, this does not always seem to be the case in the applied psychology and with other databases (Constantinescu et al., 2017). Due to the large variety of algorithms available that can lead to substantial variations in clustering solutions, we applied a double clustering strategy (explorative and confirmatory) to detect which solution was more appropriate for our data. Furthermore, participants in the present study were young students, mainly females, and highly educated. These sample characteristics potentially threat the generalizability of our findings. Further studies need to explore the presence of age and sex-related effects, (as well as cognitive styles; Carlucci et al., 2015, 2020) in ratings affective images as clustered in the present database (Fairfield et al., 2017).

In addition, the overall selection of our clusters was constrained by the valence of images selected (negative, positive, and neutral). Undoubtedly, future studies need to take into consideration arousal as well. Here, we considered arousal in our analyses but did not use it as a criterion for image selection. However, z-scores for image arousal and valence by cluster are available for use by researchers when choosing affective stimuli. These concerns could be addressed by new pictures to be included in future studies.

## Conclusion

To date, the present study represents a first attempt to provide a common stimuli metrics to which researchers could gain comparable results, since a unique and standardized database of affective stimuli based on a series of objective criteria and rigorous data analysis process were proposed. The present database, with accompanying ratings and image parameters, allows researchers to select visual stimulus materials that are independent from dimensional/discrete-category theoretical background, and to provide information on the implicit effects triggered by such stimuli.

Further studies will need to confirm the influence of cluster membership on performance and extend results to other material and cognitive tasks aimed to facilitate the sharing of a common methodology and study comparisons in aging and emotion literature. For instance, future cross-cultural studies could address the presence of common cognitive patterns or cultural differences to assess bottom-up cognitive functions.

## Data Availability Statement

The raw data supporting the conclusions of this article will be made available by the authors, without undue reservation.

## Ethics Statement

The studies involving human participants were reviewed and approved by Department of Psychological Sciences, Health and Territory, University of Chieti, Italy, Review Board. The patients/participants provided their written informed consent to participate in this study.

## Author Contributions

BF, BP, CP, and MB conceived and designed the experiments. BP and CP performed the experiments. BP and LC analyzed data. BF, BP, and LC wrote the article. All authors discussed the results and provided comments. All authors contributed to the article and approved the submitted version.

### Conflict of Interest

The authors declare that the research was conducted in the absence of any commercial or financial relationships that could be construed as a potential conflict of interest.

## Supplementary Material

The Supplementary Material for this article can be found online at: https://www.frontiersin.org/articles/10.3389/fpsyg.2020.02187/full#supplementary-material
